# Non-invasive diagnosis of retinoblastoma using cell-free DNA from aqueous humour

**DOI:** 10.1136/bjophthalmol-2018-313005

**Published:** 2019-02-11

**Authors:** Amy Gerrish, Edward Stone, Samuel Clokie, John R Ainsworth, Helen Jenkinson, Maureen McCalla, Carol Hitchcott, Isabel Colmenero, Stephanie Allen, Manoj Parulekar, Trevor Cole

**Affiliations:** 1 West Midlands Regional Genetics Service, Birmingham Women's and Children's NHS Foundation Trust, Birmingham, UK; 2 Birmingham Children’s Hospital Eye Department, Birmingham Women's and Children's NHS Foundation Trust, Birmingham, UK; 3 Department of Histopathology, Birmingham Women’s and Children’s NHS Foundation Trust, Birmingham, UK

**Keywords:** genetics, diagnostic tests/investigation, aqueous humour

## Abstract

Retinoblastoma is the most common eye malignancy in childhood caused by mutations in the *RB1* gene. Both alleles of the *RB1* gene must be mutated for tumour development. The initial *RB1* mutation may be constitutional germline or somatic (originating in one retinal cell only). Distinguishing between these alternative mechanisms is crucial, with wider implications for management of the patient and family members. Bilateral retinoblastoma is nearly always due to a constitutional mutation; however, approximately 15% of unilateral cases also carry a germline mutation, and identifying these cases is important. This can be achieved by identifying both mutation types in tumour tissue and excluding their presence in blood. Modern eye-saving chemotherapy treatment (systemic, intra-arterial and intravitreal) has resulted in fewer enucleations. As a result, tumour tissue required to identify sporadic *RB1* mutation(s) is not always available. Modern intravitreal chemotherapeutic techniques for retinoblastoma involve aspiration of aqueous humour (AH), providing a novel sample source for analysis. By analysing cell-free DNA present in the AH fluid of eyes affected with retinoblastoma, we have developed a screening test capable of detecting somatic *RB1* mutations that is comparable to current tests on enucleated tumour tissue. The results obtained with fluid from enucleated eyes were concordant with tumour tissue in all 10 cases analysed. In addition, AH analysis from two patients undergoing intravitreal chemotherapy successfully identified somatic variants in both cases. Our findings suggest that AH fluid is a promising source of tumour-derived DNA in retinoblastoma for analysis.

## Introduction

Retinoblastoma (RB) is the most common childhood eye cancer with an incidence of ~1:20 000 live births (reviewed in Lohmann and Gallie^1^). Most retinoblastomas are caused by biallelic mutations in the *RB1* gene, with non-germline cases being caused by two somatic hits, and inherited cases caused by an initial germline hit and a further somatic hit. Determining the genetic cause of RB is important for planning management of individual cases and prognostic counselling. Patients with *RB1* germline mutations are at risk of bilateral disease and non-ocular cancers. There is also the additional risk that other family members may carry the same mutation. Analysis of peripheral blood samples along with tumour DNA (tDNA) accessed through enucleation allows for the complete genetic picture to be uncovered. The possibility that a patient has inherited RB can only be discounted by the detection of two mutations within the tumour sample, which are not present in the blood. Increased treatment success with modalities such as systemic or locally delivered chemotherapy suggests that tDNA is often unavailable rendering it impossible to identify somatic *RB1* mutations.^2 3^

The existence of cell-free DNA (cfDNA) in peripheral blood was noted in 1989^4^ and has since been utilised for many applications including cancer detection (reviewed in Stewart and Tsui^5^) as well as for non-invasive prenatal diagnostics (reviewed in Wong and Lo^6^). We hypothesised that intraocular fluid removed during intravitreal chemotherapy (IVC) treatment would be a source of cell-free tumour DNA and therefore useful for detection of somatic variants in patients with retinoblastoma and other ocular disorders. This publication reports our findings using next-generation sequencing (NGS). Together with the recently published study of Berry *et al*,^7^ this demonstrates that aqueous humour (AH) can be used as a surrogate biopsy for analysis of tumour-derived DNA in retinoblastoma eyes to monitor somatic variants.

## Materials and methods

### Sample collection and processing

Following ethical approval, subjects were recruited through the national referral retinoblastoma unit. Approximately 100 μL of AH fluid was taken from chemonaive primary enucleated eyes by anterior chamber tap prior to opening the eye for tumour dissection, or prior to injection during IVC treatment in eyes with recurrent disease following prior systemic chemotherapy and local treatment. Venous blood was collected at the same time for genetic testing.

### Targeted massively parallel sequencing (MPS) and bioinformatic analysis

DNA extracted from blood, AH fluid and tumour (enucleated samples only) was sequenced by MPS following targeted capture enrichment for the promoter and exonic regions of the *RB1* gene (NM_000321; chr13: 48,877,549–49,056,076, hg19), highly heterozygous single nucleotide polymorphisms and non-polymorphic regions across chromosome 13. Bioinformatic analysis performed for single-nucleotide variant (SNV), copy number variant and loss of heterozygosity (LOH) detection using an in-house pipeline (information available on request).

## Results

### Evaluation of cfDNA in AH of patients with retinoblastoma

**Figure 1:**
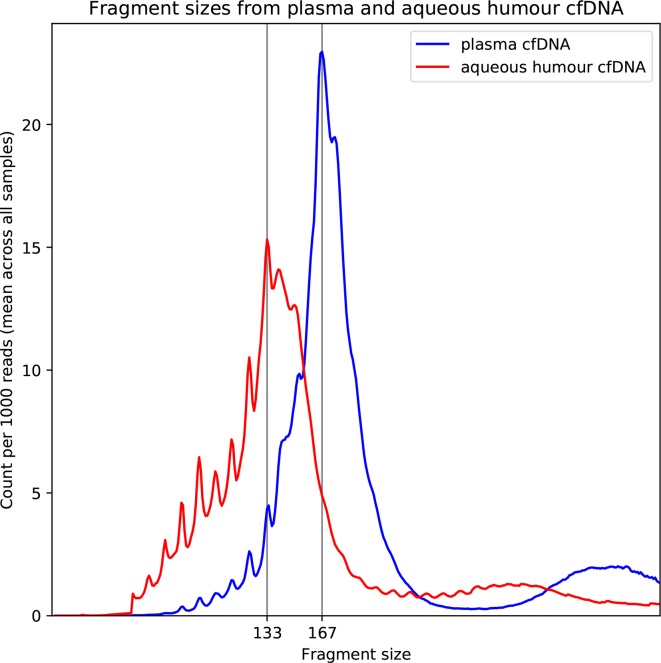
Average cell-free DNA (cfDNA) fragment size post next-generation sequencing of 12 aqueous humour samples (red) and five plasma samples (blue) normalised to read depth. Position of peaks has been marked.

AH samples were obtained from 10 eyes post enucleation and from two patients with retinoblastoma undergoing IVC treatment. cfDNA concentrations from the enucleated eyes were found to be highly variable (0.14–394 ng/μL, median 1.67 ng/μL). The AH samples from the patients treated with intravitreous injection had lower concentrations of cfDNA and could not be accurately quantified (<0.100 ng/μL); however, we were able to detect the presence of cfDNA. The DNA size distribution peaked at 133 bp in comparison to the plasma cfDNA which peaked at 167 bp (confirmed by bioinformatic analysis of NGS data (figure 1)). Previous studies have reported that tumour-derived cfDNA is shorter than plasma cfDNA,^8 9^ suggesting that the cfDNA detected within our AH samples is of tumour origin.

### Variant detection in cfDNA from enucleated eyes of patients with retinoblastoma

**Figure 2:**
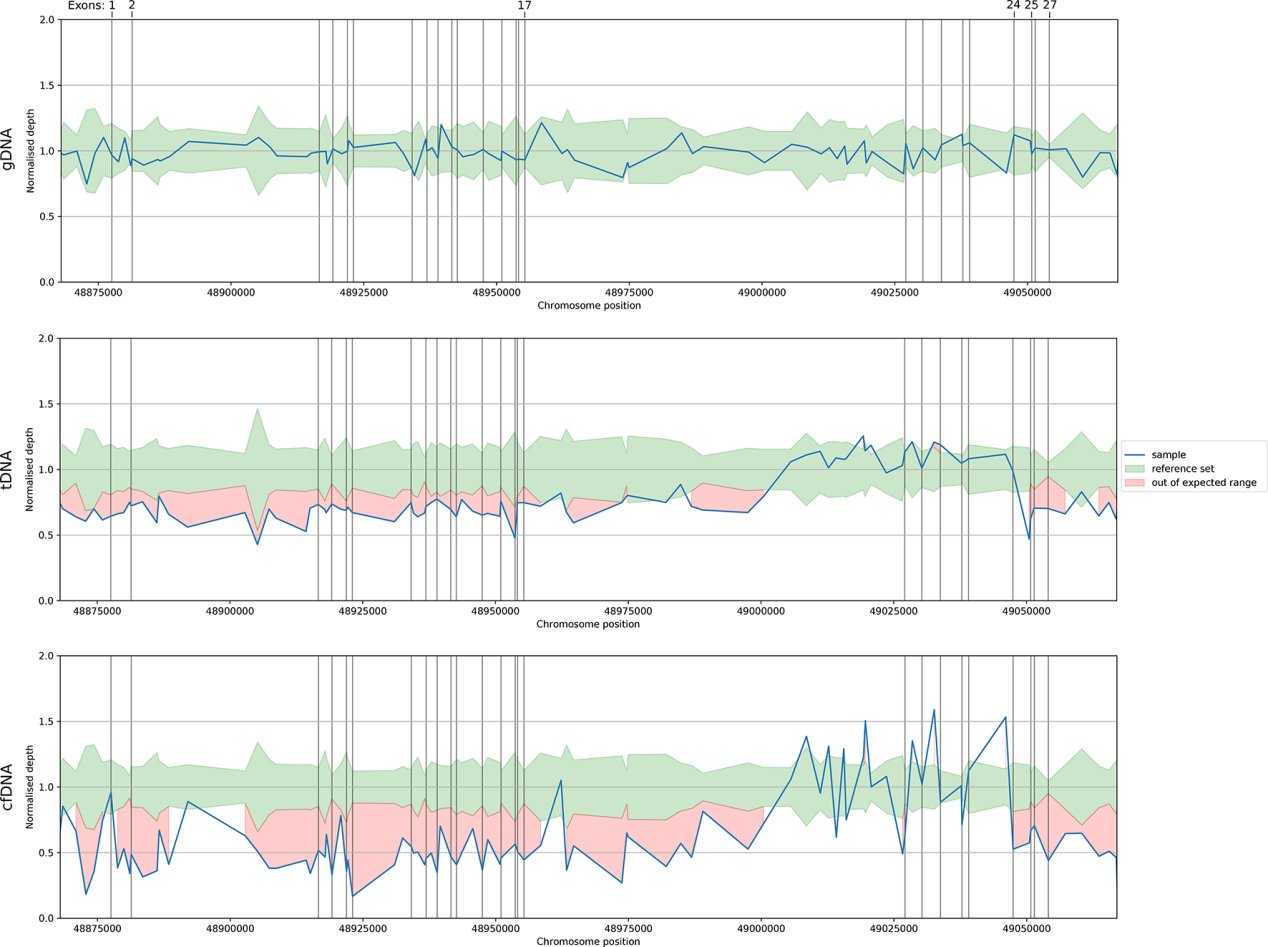
Example of copy number variant (CNV) analysis within the genomic DNA (gDNA), tumour DNA (tDNA) and cell-free (cfDNA) of sample E6 showing the detection of two large *RB1* deletions in both the tDNA and cfDNA. CNVs are detected by comparing the relative depth of coverage achieved at each target site within the sample against a reference set of normal controls. Vertical lines indicate the positions of the target sites capturing the *RB1* exons.

In order to validate our assay, we sequenced cfDNA and tDNA extracted from 10 enucleated eyes. Analysis of the tDNA from the enucleated eyes identified 11 *RB1* SNVs, two large *RB1* deletions and seven regions of LOH (table 1). This concurred with the previous diagnostic testing results. Analysis of genomic DNA (gDNA) determined that two of these variants were germline mosaic at 9% and 21%, respectively. These results confirmed that our NGS assay can detect a variety of *RB1* mutations at varying levels. We then attempted to detect these mutations in cfDNA extracted from the AH. In all but one case, mutation results from cfDNA were fully concordant with results obtained from tumour tissue (table 1). In the remaining patient, while we were able to detect the two large *RB1* exonic deletions present in the tumour, the start point of each deletion was discordant between the two sample types (figure 2). Interestingly, in all cases, the mutant *RB1* allele frequency was shown to be >90% in the cfDNA where one allele of *RB1* remained (LOH cases) or >45% where two *RB1 alleles* (one mutant and one wild type) were present, suggesting that >90% of the cfDNA present in AH of enucleated eyes is of tumour origin. This result supports our size profile results.

**Table 1:** MPS results for enucleated (E1–10) and IVC (IVC1–2) samples

Patient	cfDNA conc (ng/μL)	RB1 mutation	gDNA	tDNA	cfDNA
			% mutation	Result /% mutation	Result /% mutation
E1	2.12	c.1363C>T p.(Arg455*)		76	91
		LOH		Complete LOH	Complete LOH
E2	228	c.751C>T p.(Arg251*)		91	99
		LOH		Complete LOH	Complete LOH
E3	0.183	c.1959dupA		76	87
		LOH		Partial LOH	Partial LOH
E4	394	c.763C>T p.(Arg255*)		99	90
		LOH		Partial LOH	Partial LOH
E5	0.169	c.1251_1252delAA		91	94
		LOH		Partial LOH	Partial LOH
E6	0.141	Deletion exons 1–17		Deletion exons 1–17	Deletion exons 2–17
		Deletion exons 25–27		Deletion exons 25–27	Deletion exons 24–27
E7	244	c.1496_97dup p.Arg500Alafs*20	8	90	94
		LOH		Partial LOH	Partial LOH
E8	1.96	c.1072 C>T p.(Arg358*)	21†	97	100
		LOH		Partial LOH	Partial LOH
E9	1.01	c.958C>T p.(Arg320*)		43	44
		c.1981C>T p.(Arg661*)		46	46
E10	1.38	c.147delT		38	46
		c.1330C>T p.(Gln444*)		50	44

IVC 1	<0.100	c.751C>T p.(Arg251*)†		N/A N/A	49
		c.1654C>T (Arg552*)	45		58
IVC 2	<0.100	LOH†			Partial LOH
		c.2490–1_2490delGA	54		96

Results are shown as percentage of mutation sequencing reads in gDNA, tDNA and cfDNA where appropriate. Complete LOH corresponds to LOH of whole chromosome 13; partial LOH indicates LOH of parts of chromosome 13, encompassing 13q14.

† Previously undetected

AH, aqueous humour; IVC, intravitreal chemotherapy; LOH, loss of heterozygosity; MPS, massively parallel sequencing; RB, retinoblastoma; cfDNA, cell-free DNA; gDNA, genomic DNA; tDNA, tumour DNA.

### Variant detection in cfDNA from patients undergoing IVC

As a proof of principle, we sequenced cfDNA from two IVC samples. Diagnostic testing had previously identified one germline mutation in each patient but the somatic variants were unknown due to lack of tDNA. Germline mutations in both gDNA and cfDNA samples were detected in addition to the somatic variants in AH cfDNA samples (table 1). These were SNV c.751C>T p.(Arg251*) in patient 1 and a region of LOH (chr 13; 23,045,500–115,169,878) in patient 2. The mutation allele frequency of these variants showed a similar pattern to the enucleated cfDNA samples, suggesting that the majority of the cfDNA in IVC samples is also derived from the tumour.

## Discussion

The introduction of new techniques in the conservative management of retinoblastoma, including IVC, has dramatically reduced enucleation rates.

This study demonstrates the feasibility of testing AH samples in non-enucleated eyes, with AH cfDNA enabling detection of somatic variants in patients undergoing IVC where tumour is not available. Capture-based technology was used to identify previously undetected *RB1* gene mutations and LOH alterations in AH cfDNA. This extends the findings of Berry *et al* and demonstrates the clinical utility of our approach although further development is required to increase the accuracy in detecting larger exon-spanning *RB1* mutations. Our data also suggests that the majority of cfDNA in the AH is from the tumour due to the compartmentalised nature of ocular fluids, a factor which facilitates this test.

Among the enucleated eyes tested, higher levels of cfDNA were seen in eyes undergoing primary enucleation. Adequate but lower DNA levels were present in eyes treated with systemic chemotherapy and local treatment such as laser and cryotherapy. This would suggest the cfDNA load varies with the tumour load.

The distinction between germline and somatic mutations is vital, as germline cases need close monitoring with short-term risk of new ocular tumours and long-term risk of second systemic cancers. The risks of both are greatly reduced if there is evidence to indicate a constitutional mutation is highly unlikely. Furthermore, there are important implications for screening examinations in the wider family (siblings and offspring). Confirming the non-heritable nature of the retinoblastoma can avoid screening examinations in many family members, with significant cost savings and avoid potential morbidity in unaffected family members, previously screened unnecessarily.

In view of the extensive safety record of carefully performed intraocular procedures in eyes with retinoblastoma (3 10; Munier, personal communication), the results of our study, and that of Berry *et al*, there appears to be a clear clinical benefit of diagnostic AH taps in apparent non-genetic retinoblastoma where the affected eye is retained.
