# Correction: Non-invasive diagnosis of retinoblastoma using cell-free DNA from aqueous humour

**DOI:** 10.1136/bjophthalmol-2018-313005corr1

**Published:** 2019-11-01

Gerrish A, Stone E, Clokie S, *et al*. Non-invasive diagnosis of retinoblastoma using cell-free DNA from aqueous humour (*Br J Ophthalmol* 2019;103:721–4).

The authors have noticed that because of a sample categorisation error prior to sequencing, the RB1 mutations reported for two samples in Table 1 are incorrect. This error also affected some of the data in the paragraph under the heading ‘Variant detection in cfDNA from patients undergoing IVC.’ This is the corrected paragraph: As a proof of principle, we sequenced cfDNA from two IVC samples. Diagnostic testing had previously identified a germline mutation in patient 1 but the remaining somatic variant in patient 1 and the two somatic variants in patient 2 were unknown due to lack of tDNA. The germline mutation was detected in both the gDNA and cfDNA samples of patient 1. In addition, three somatic variants were identified in the AH cfDNA samples (table 1). These were complete LOH of chromosome 13 in patient 1 plus SNV c.1078dupA p.(Ser360Lysfs*2) and a region of LOH (chr 13; 40,803,985–115,064,542) in patient 2. The mutation allele frequency of these variants showed a similar pattern to the enucleated cfDNA samples, suggesting that the majority of the cfDNA in IVC samples is also derived from the tumour.

**Table 1:** Mps results for enucleated (E1–10) and IVC (IVC1–2) samples

Patient	cfDNA conc (ng/ul)	RB1 mutation	gDNA	tDNA	cfDNA
			% mutation	Result / % mutation	Result / % mutation
E1	2.12	c.1363C>T p.(Arg455*)		76	91
		LOH		Complete LOH	Complete LOH
E2	228	c.751C>T p.(Arg251*)		91	99
		LOH		Complete LOH	Complete LOH
E3	0.183	c.1959dupA		76	87
		LOH		Partial LOH	Partial LOH
E4	394	c.763C>T p.(Arg255*)		99	90
		LOH		Partial LOH	Partial LOH
E5	0.169	c.1251_1252delAA		91	94
		LOH		Partial LOH	Partial LOH
E6	0.141	Deletion exons 1–17		Deletion exons 1–17	Deletion exons 2–17
		Deletion exons 25–27		Deletion exons 25–27	Deletion exons 24–27
E7	244	c.1496_97dup p.Arg500Alafs*20	8	90	94
		LOH		Partial LOH	Partial LOH
E8	1.96	c.1072 C>T p.(Arg358*)	21†	97	100
		LOH		Partial LOH	Partial LOH
E9	1.01	c.958C>T p.(Arg320*)		43	44
		c.1981C>T p.(Arg661*)		46	46
E10	1.38	c.147delT		38	46
		c.1330C>T p.(Gln444*)		50	44
IVC 1	<0.100	LOH†		N/A	Complete LOH
		c.1666C>T p.(Arg556*)	39		100
IVC 2	<0.100	LOH†		N/A	Partial LOH
		c.1078dupA p.(Ser360Lysfs*2)†			80

Results are shown as percentage of mutation sequencing reads in gDNA, tDNA and cfDNA where appropriate. Complete LOH corresponds to LOH of whole chromosome 13; partial LOH indicates LOH of parts of chromosome 13, encompassing 13q14.

†Previously undetected.

AH, aqueous humour; IVC, intravitreal chemotherapy; LOH, loss of heterozygosity; MPS, massively parallel sequencing; RB, retinoblastoma; cfDNA, cell-free DNA; gDNA, genomic DNA; tDNA, tumour DNA.

